# Efficacy of intraosseous access for trauma resuscitation: a systematic review and meta-analysis

**DOI:** 10.1186/s13017-023-00487-7

**Published:** 2023-03-14

**Authors:** Dong Wang, Lei Deng, Ruipeng Zhang, Yiyue Zhou, Jun Zeng, Hua Jiang

**Affiliations:** 1 Institute for Emergency and Disaster Medicine, Sichuan Provincial People’s Hospital, University of Electronic Science and Technology of China, No. 32, Yi Huan Lu Xi Er Duan, Chengdu, 610072 Sichuan Province China; 2 Chinese Academy of Sciences, Sichuan Translational Medicine Research Hospital, Chengdu, 610072 China; 3 Sichuan Province Clinical Research Center for Emergency and Critical Care Medicine, Sichuan Provincial People’s Hospital, University of Electronic Science and Technology of China, Chengdu, 610072 China; 4 Department of Biology, Sorbonne University, 75005 Paris, France

**Keywords:** Intraosseous access, Intravenous access, Trauma, Pre-hospital care, Resuscitation

## Abstract

**Background:**

During medical emergencies, intraosseous (IO) access and intravenous (IV) access are methods of administering therapies and medications to patients. Treating patients in emergency medical situations is a highly time sensitive practice; however, research into the optimal access method is limited and existing systematic reviews have only considered out-of-hospital cardiac arrest (OHCA) patients. We focused on severe trauma patients and conducted a systematic review to evaluate the efficacy and efficiency of intraosseous (IO) access compared to intravenous (IV) access for trauma resuscitation in prehospital care.

**Materials and method:**

PubMed, Web of Science, Cochrane Library, EMBASE, ScienceDirect, banque de données en santé publique and CNKI databases were searched for articles published between January 1, 2000, and January 31, 2023. Adult trauma patients were included, regardless of race, nationality, and region. OHCA patients and other types of patients were excluded. The experimental and control groups received IO and IV access, respectively, in the pre-hospital and emergency departments for salvage. The primary outcome was success rate on first attempt, which was defined as secure needle position in the marrow cavity or a peripheral vein, with normal fluid flow. Secondary outcomes included mean time to resuscitation, mean procedure time, and complications.

**Results:**

Three reviewers independently screened the literature, extracted the data, and assessed the risk of bias in the included studies; meta-analyses were then performed using Review Manager (Version 5.4; Cochrane, Oxford, UK). The success rate on first attempt was significant higher for IO access than for IV access (RR = 1.46, 95% CI [1.16, 1.85], *P* = 0.001). The mean procedure time was significantly reduced (MD = − 5.67, 95% CI [− 9.26, − 2.07], *P* = 0.002). There was no significant difference in mean time to resuscitation (MD = − 1.00, 95% CI [− 3.18, 1.17], *P* = 0.37) and complications (RR = 1.22, 95% CI [0.14, 10.62], *P* = 0.86) between the IO and IV groups.

**Conclusion:**

The success rate on first attempt of IO access was much higher than that of IV access for trauma patients, and the mean procedure time of IO access was significantly less when compared to IV access. Therefore, IO access should be suggested as an urgent vascular access for hypotensive trauma patients, especially those who are under severe shock.

**Supplementary Information:**

The online version contains supplementary material available at 10.1186/s13017-023-00487-7.

## Introduction

Intravenous (IV) administration is an important aspect of clinical practice; however, it can be challenging in the prehospital environment when a patient enters a state of shock causing the blood vessels to collapse making venipuncture difficult. In such cases, intraosseous (IO) access is an alternative approach [1].

Since IO was invented by Drinker in 1916 [2], it has been a controversial and doubtful approach. The advantage of IO was that it can shorten the time to obtain vascular access. In recent decades, intraosseous devices have been updated and introduced to emergency and pre-hospital services [3].IO access can be used in many conditions such as severe trauma, out-of-hospital cardiac arrest (OHCA), war injuries, and so on.

Updated guidelines by American Heart Association (AHA) considers intraosseous (IO) access an acceptable vascular access equal to intravenous (IV) access [4], while the European Resuscitation Council guidelines recommended shifting to IO access immediately if IV access failed after 1 min [5]. Despite these recommendations, a study of American emergency medicine (EM) residency training programs demonstrated that IO access was only considered the fourth choice in unstable patients requiring emergent vascular access [6]. In addition, several studies suggested that IO is infrequently used in adult resuscitations [7–9]. Some studies have shown that IO access did not improve survival outcomes when compared with IV access. In recent years, several systematic reviews (SRs) have been published to evaluate the efficiency of IO access, however, the aims of these SRs only focus on OHCA patients. So far, there is no meta-analysis to compare the outcomes of IO and IV in trauma patients. Since IO is suitable for resuscitation of various diseases and it is easier to obtain venous access, we speculate that it should play a role in trauma resuscitation.

Therefore, we conducted this systematic review and meta-analysis about trauma patients to provide comprehensive evidence for clinical practitioners and researchers. We aim compare the efficacy and efficiency of IO access with those of IV access used for trauma resuscitation in the emergency department and prehospital care.

## Material and methods

We followed the Preferred Reporting Items for Systematic Reviews and Meta-analyses (PRISMA) reporting guidelines for this review [10]. The registration number is CRD42022299317.

### Criteria for selecting studies

#### Inclusion criteria

(i) *Participants* We included the experiments with adult trauma and shock patients, regardless of their race, nationality, and region.
(ii) *Interventions* The experimental group received IO access in pre-hospital and emergency department for salvage.
(iii) *Comparisons* The control group received IV access in pre-hospital and emergency department for salvage.

#### Exclusion criteria

(i) Duplicated literature.
(ii) Animal experiments, case reports, conference papers, cadaveric experiments, and before-and-after studies.
(iii) Studies not reporting the outcomes and lack comparison.

Types of studies: randomized controlled trials (RCTs), cohort studies, and case–control studies.

Primary outcome: Success rate on first attempt, which is defined as secure needle position in the marrow cavity or a peripheral vein, with normal fluid flow.

#### Secondary outcomes

(i) *Mean time to resuscitation* Time to resuscitation was measured from the establishment of intravenous or intraosseous access to the recovery of blood pressure of patients.
(ii) *Mean procedure time* Intravenous procedure time was measured from the opening of the kit to the aspiration of venous blood. For intraosseous placement, procedure time was measured from the opening of the kit to when marrow aspirates were confirmed from the attached tubing.
(iii) *Complications* Complications include symptom s during operation, such as exfoliation, as well as symptoms after operation, such as infection, extravasation, soft tissue necrosis, and so on.

### Literature retrieval and identification of studies

PubMed, Web of Science, Cochrane Library, EMBASE, ScienceDirect, banque de données en santé publique (BDSP) and CNKI databases were searched for articles published from January 1, 2000, to January 31, 2023. Terms were searched in medical subheading (MeSH) and free terms in PubMed. Retrieval terms include “infusions, intraosseous,” “resuscitation,” “emergency,” “emergency care, prehospital,” “Wounds and Injuries,” and “Multiple Trauma.” Chinese terms include “gu shu ye,” “gu sui shu ye,” “gu shu ye tong dao", “gu sui shu ye ji shu,” “chuang shang,” “xiu ke” and “ji zhen.” French terms include “perfusion intraosseuse,” “réanimation,” “soins d'urgence” and “préhospitalier.” We also reviewed the references of the included articles and related systematic reviews to identify additional relevant studies. The search strategy is shown in Supplementary material.

### Selection of studies and data extraction

Three reviewers (WD, ZRP and ZYY) independently performed the literature retrieval, screening, data extraction, and quality evaluation. Any discrepancies between the findings were resolved by a fourth reviewer (DL). Data extraction included the baseline data of the enrolled patients, intervention, comparison, success rate of first attempt, mean time to resuscitation, mean procedure time, and complications.

### Quality assessment

The Newcastle–Ottawa scale (NOS) was used to evaluate the quality of the observational studies. Studies with scores higher than or equal to five points were considered high quality. The modified Jadad’s score was used to evaluate the RCT quality. Studies that scored higher than four points were considered high quality. Two reviewers (WD and ZRP) independently rated the risk of bias of randomized controlled trials using the Cochrane risk of bias. Version 1 (RoB-1 tool). Disagreements were resolved by consensus. The ROBINS-I tool was used to assess the risk of bias in non-RCT studies.

### Certainty of evidence

Certainty of evidence was determined by the Grading of Recommendations Assessment, Development, and Evaluation (GRADE) approach using the GRADE Pro online website tools. This was assessed by two independent reviewers (WD and ZRP) [11]. In accordance with the study design, study quality, precision, consistency, directness, and risk of reporting bias, we assessed the quality of evidence and confidence in the effect estimates. The overall quality of evidence was described as “high,” “moderate,” “low,” or “very low” for each outcome in Table 1.

**Table 1:** Certainty assessment of evidence

Certainty assessment							No of patients		Effect		Certainty	Importance
№ of studies	Study design	Risk of bias	Inconsistency	Indirectness	Imprecision	Other considerations	Intraosseous access	intravenous access	Relative (95% CI)	Absolute (95% CI)		
*Success rate on first attempt*												
5	Randomized trials	Not serious	Serious	Not serious	Not serious	None	168/185 (90.8%)	141/232 (60.8%)	RR 1.48 (1.32 to 1.66)	292 more per 1000 (from 194 to 401 more)	⨁⨁⨁◯ Moderate	CRITICAL
*Mean time to resuscitation*												
4	Randomized trials	Not serious	Serious	Not serious	Not serious	None	239	253	–	MD 1.26 lower (1.57 lower to 0.94 lower)	⨁⨁⨁◯ Moderate	IMPORTANT
*Mean procedure time*												
5	Randomized trials	Not serious	Serious	Not serious	Not serious	None	279	293	–	MD 2.58 lower (2.78 lower to 2.37 lower)	⨁⨁⨁◯ Moderate	IMPORTANT
*Complications*												
4	Randomized trials	Serious	Serious	Not serious	Not serious	All plausible residual confounding would reduce the demonstrated effect	23/139 (16.5%)	34/186 (18.3%)	RR 0.97 (0.61 to 1.55)	5 fewer per 1,000 (from 71 fewer to 101 more)	⨁⨁⨁◯ Moderate	IMPORTANT

*CI* Confidence interval, *MD* mean difference, *RR* Risk ratio

### Statistical analyses

The Mantel–Haenszel method was used to obtain the risk ratio (RR) and 95% confidence interval (CI) for dichotomous variables. The inverse variance method was used to obtain the mean difference (MD) and 95% CI for continuous variables. The test level was set at *α* = 0.05. Statistical significance was set at *P* < 0.05. Statistical heterogeneity was evaluated using *I*^2^ (*I*^2^ > 50%, indicating an elevated level of heterogeneity). Random-effect models were used when heterogeneous results appeared (*I*^2^ ≠ 0) [12]. Otherwise, a fixed-effects model was used. In addition, subgroup analysis was performed according to patient type. Publication bias was evaluated when the number of included studies was more than ten because when there were fewer studies, the power of the tests was low [11]. Sensitivity analysis was performed to examine the stability of the results. Data analysis was performed using Review Manager (Version 5.4; Cochrane, Oxford, UK).

## Results

### Literature retrieval results and study characteristics

**Fig. 1:**
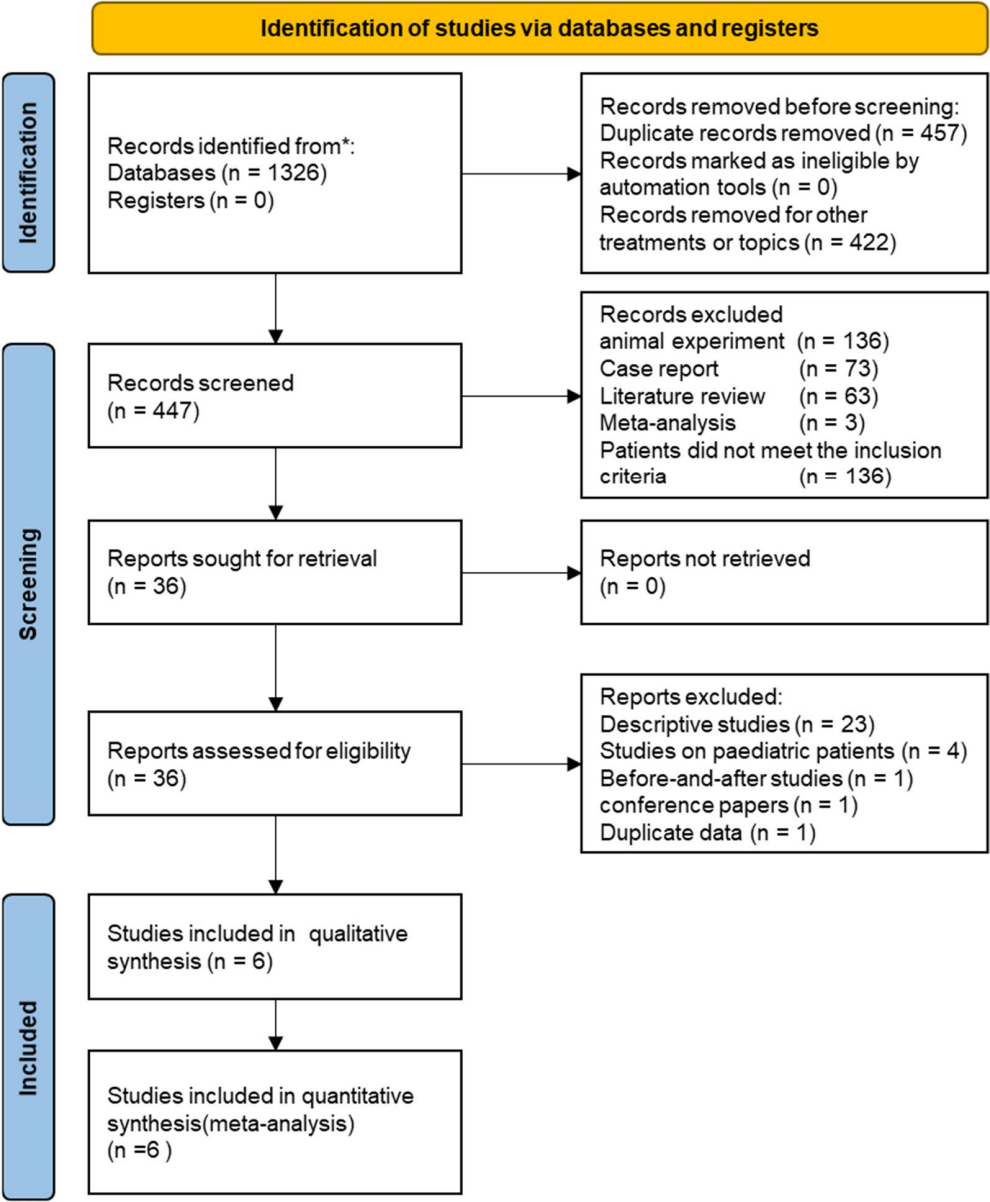
Identification of studies from databases and registers

Through literature retrieval, using the strategy we reported above, 1326 records were found. Forty-four articles on this subject were found in CNKI and ninety-six articles were found in BDSP. After screening and evaluation, eight studies [13–20] were finally included in the qualitative analysis and the meta-analysis. Identification of studies from databases and registers are shown in Fig. 1. The eligible studies were published during 2007–2022. These were from the USA (2) and China (6). Three articles were cohort studies. In total, 291 patients were enrolled. Five were RCTs involving 548 patients. Details of the baseline information of the included studies are presented in Table 2.

**Table 2:** Summary of detailed information about the included studies

Author, year	Study design	Research period	Country	Patient type	Sample size Total (I/C)	Age (years) Total (I/C)	Sex (Male%)
Jixin et al. [17]	RCT	2001.01–2006.05	China	Hemorrhagic shock	268 (127/141)	16–45 (/)	100% 100%/100%
James et al. [14]	Prospective cohort study	2008.6.15–2008.8.15	USA	Shock	92 (30/62)	/ 46.9/56.2/50.8	/ 63.3%/53.2%
Yun et al. [16]	Retrospective cohort study	2008.1–2011.6	China	Traffic injury	120 (60/60)	39 ± 12 (/)	70.10% (/)
Chong et al. [15]	RCT	2006.01–2008.01	China	Trauma and shock	32 (16/16)	/ 33.9 ± 5.3/33.1 ± 6.0	61.70% (/)
Peter et al. [18]	Prospective cohort study	2012.2–2013.7	USA	MET calls	79 (31/48)	/	/
Yan-yan et al. [13]	RCT	2017.4.1–2018.12.31	China	Shock	96 (48/48)	20–95 (65.6 ± 17.1)	65.63% /
Yinxue et al. [19]	RCT	2018.12–2020.10	China	Hemorrhagic shock caused by trauma and burn	80 (40/40)	/ 53.1 ± 3.4/53.2 ± 3.3	66.3% 67.5%/65.0%
Yin-e et al. [20]	RCT	2019.9–2021.5	China	Hemorrhagic shock caused by trauma and traffic injury	72 (36/36)	/ 39.5 ± 4.2/40.2 ± 5.6	56.9% 55.6%/58.3%

*IQR* Interquartile range, *RCT* Randomized controlled trial, *USA* United States, *I/C* intervention group/comparison group

### Quality assessment

In total of eight studies meet inclusion criteria and are enrolled. Three are retrospective studies, and the NOS scores ranged from 4 to 7, indicating that the methodological quality was fair. Five are RCTs. According to the criteria of the modified Jadad’s scores, two studies were considered high quality, while three studies were considered low quality. The details of the quality evaluation of each study are presented in Tables 3 and 4.

**Table 3:** Newcastle–Ottawa Quality assessment scale for cohort studies

Study	Selection			Comparability			Outcome		Total
	Representativeness of the exposed cohort	Selection of the non-exposed cohort	Ascertainment of exposure	Demonstration that outcome of interest was not present at the start of the study	Comparability of cohorts on the basis of the design or analysis	Assessment of outcome	Was follow up long enough for outcomes to occur	Adequacy of follow-up of cohorts	
	*	*	*	*	**	*	*	*	**** *****
Peter et al. [18]	*	*	*	*		*			*****
James et al. [14]	*	*	*	*		*			*****
Yun et al. [16]	*	*	*		*	*			*****

**Table 4:** Modified Jadad’s scores scale for randomized controlled trials

	Randomization	Concealment	Blinded	With or drop-out	Total
Jixin et al. [17]	2	1	0	1	4
Chong et al. [15]	1	0	0	0	1
Yan-yan et al. [13]	2	2	1	0	5
Yinxue et al. [19]	2	1	0	0	3
Yin-e et al. [20]	2	1	0	0	3

**Fig. 2:**
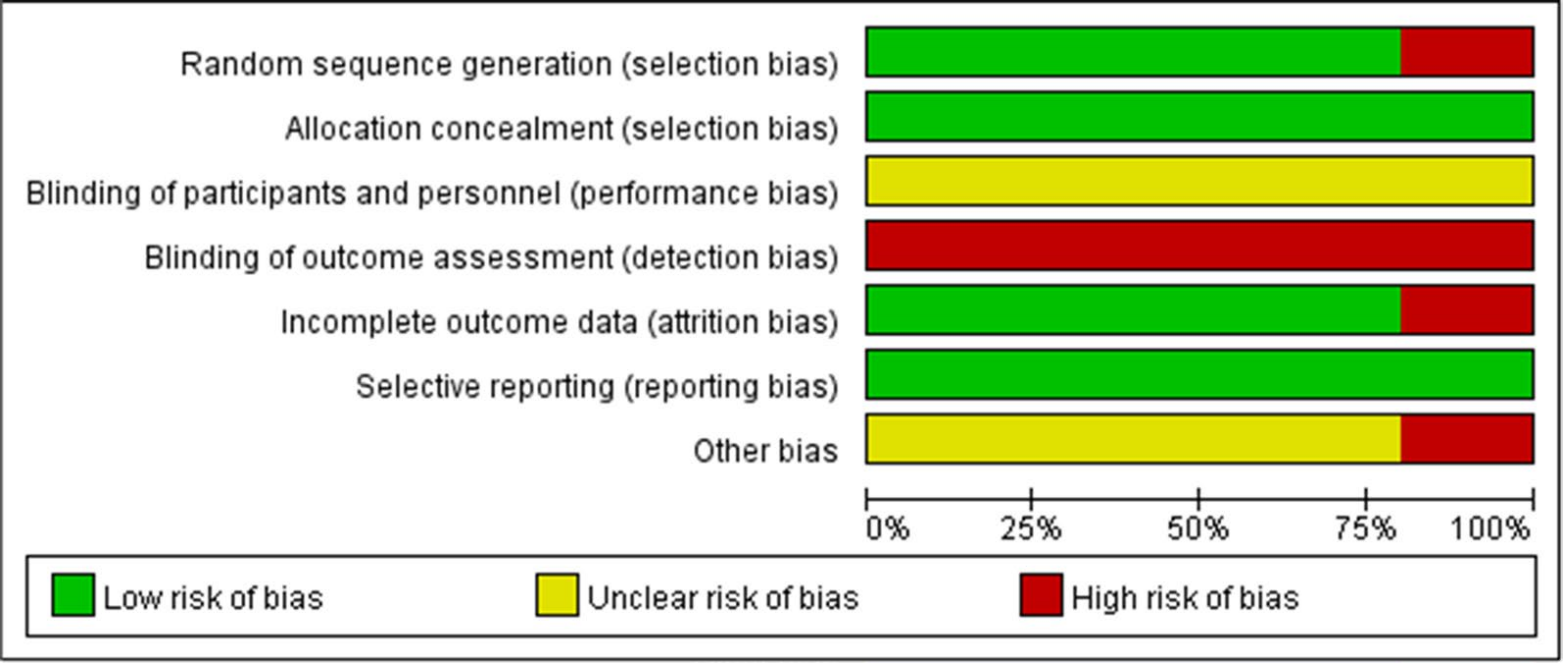
The risk of bias graph for randomized controlled trials based on ROB-1

**Fig. 3:**
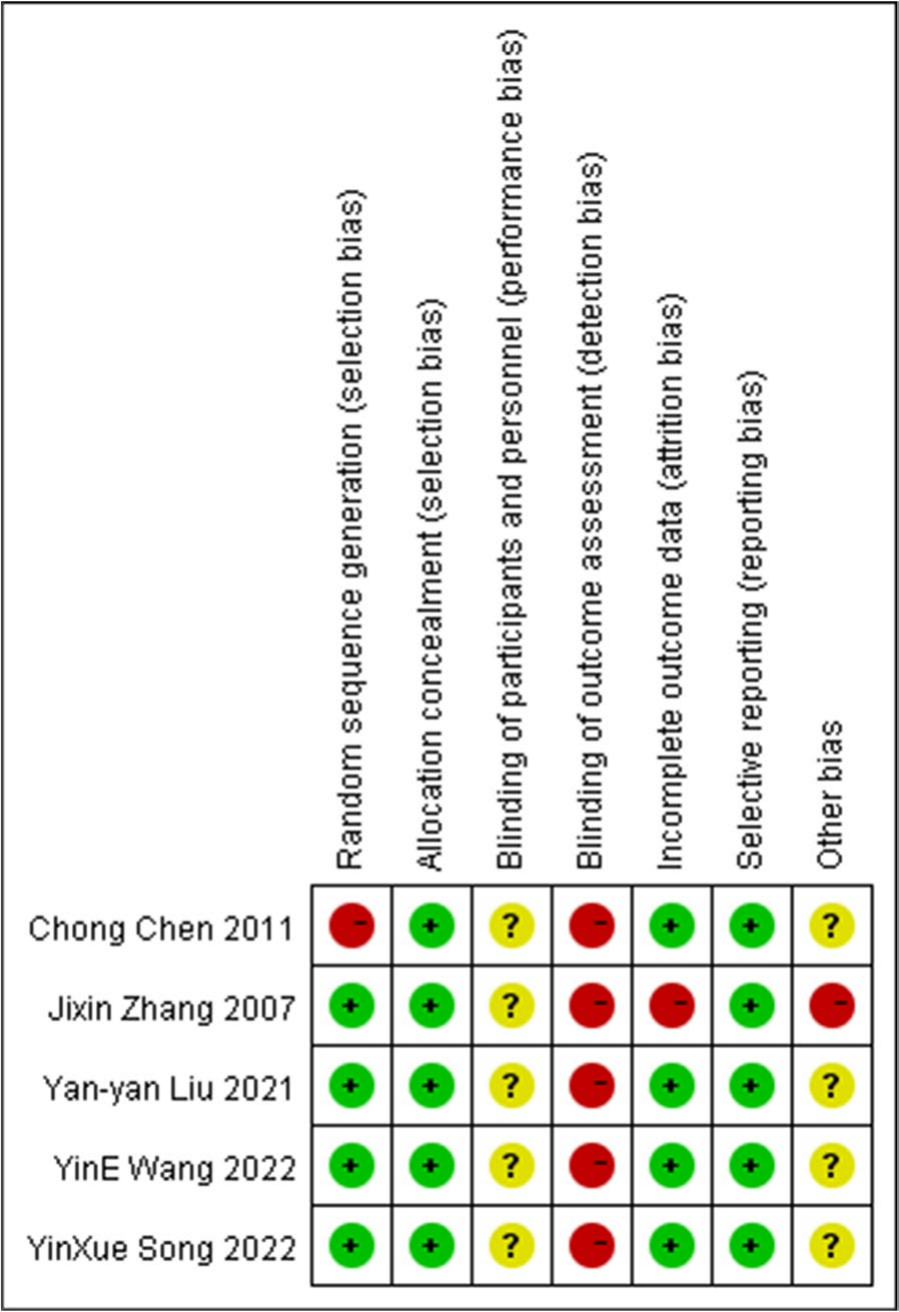
The risk of bias summary for randomized controlled trials based on ROB-1

**Fig. 4:**
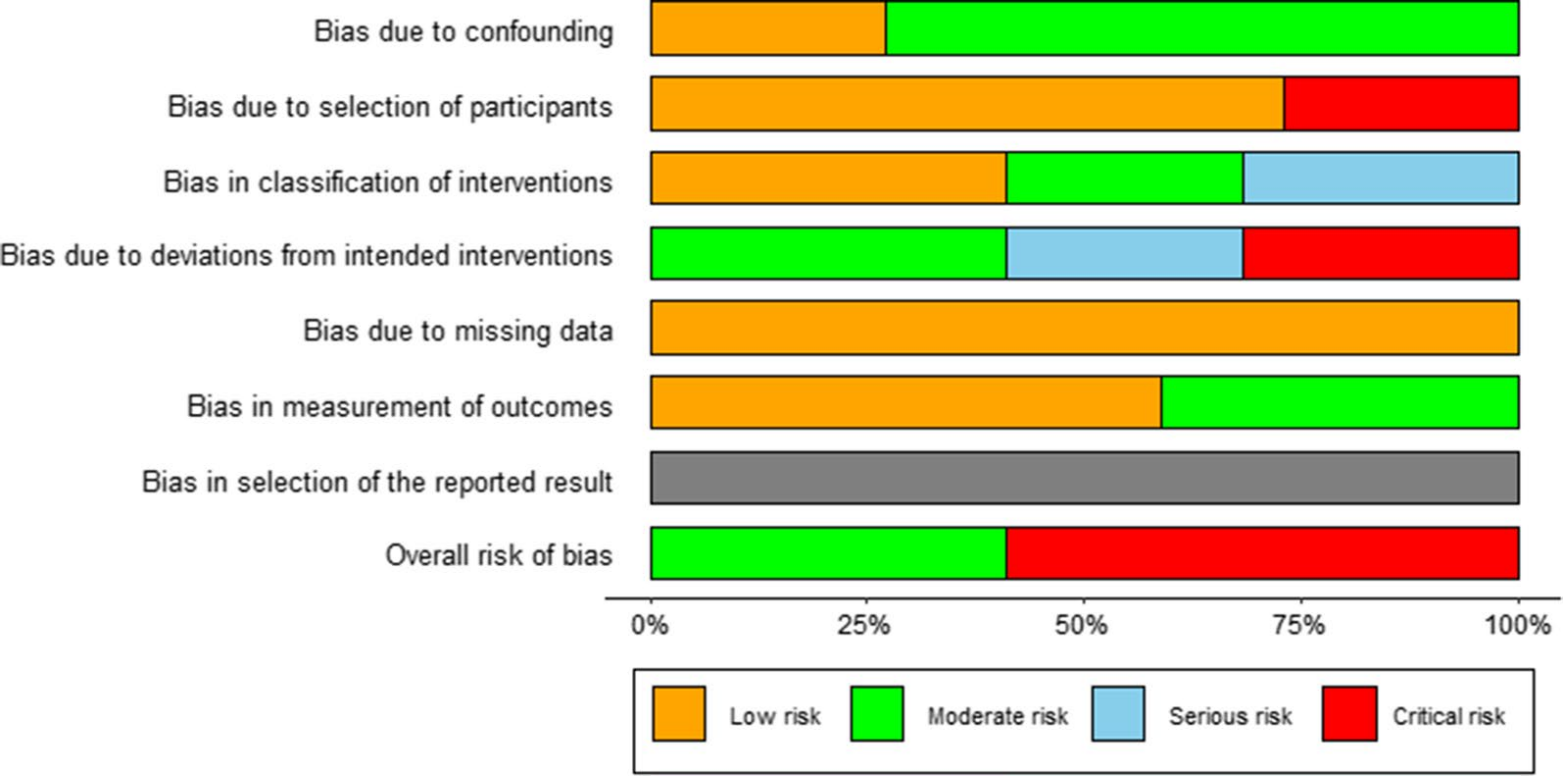
The risk of bias graph for cohort studies based on ROBINS-I

ROB-1 indicates high risk for five studies. Because different equipment was used in the studies, researchers could not follow the blind method of participants. The risk of bias graph and risk of bias summary are shown in Figs. 2 and 3. ROBINS-I shows critical risk for two studies and moderate risk for one study. The details are presented in Fig. 4.

### Primary outcome

#### Success rate on first attempt

**Fig. 5:**
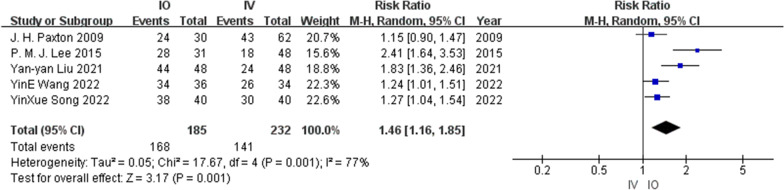
Success rate on first attempt

Three RCT [13, 19, 20] and two prospective cohort studies [14, 18] reported first-pass success rates involving 185 patients in the IO group and 232 patients in the IV group. Heterogeneity (*I*^2^ = 77%) was observed among the studies, and a random-effects model was used. Cumulative analysis showed that the success rate on first attempt in the IO group was higher than that in the IV group (RR = 1.46, 95% CI [1.16, 1.85], *P* = 0.001). Figure 5 shows a forest plot of the success rate on the first attempt.

### Secondary outcome

#### Mean time to resuscitation

**Fig. 6:**
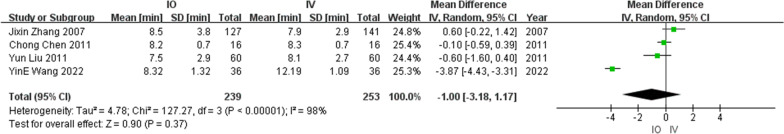
Mean time to resuscitation

Three RCTs [15, 17, 20] and one retrospective cohort studies [16] reported the mean time to resuscitation involving 239 patients in the IO group and 253 in the IV group. Heterogeneity (*I*^2^ = 98%) was observed among the studies, and a random-effects model was used. Cumulative analysis showed no difference between the IO and IV group (MD = − 1.00, 95% CI [− 3.18, 1.17], *P* = 0.37). Figure 6 shows a forest plot of the mean time to resuscitation.

#### Mean procedure time

**Fig. 7:**
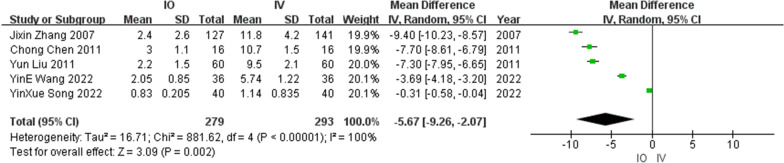
Mean procedure time

Four RCTs [15, 17, 19, 20] and one retrospective cohort studies [16] reported the mean procedure times involving 279 patients in the IO group and 293 patients in the IV group. Heterogeneity (*I*^2^ = 100%) was observed among the studies, and a random-effects model was used. Cumulative analysis showed that the interval in the IO group tended to be shorter than that in the IV group (MD = − 5.67, 95% CI [− 9.26, − 2.07], *P* = 0.02). Figure 7 shows a forest plot of the mean procedure time.

#### Complications

**Fig. 8:**
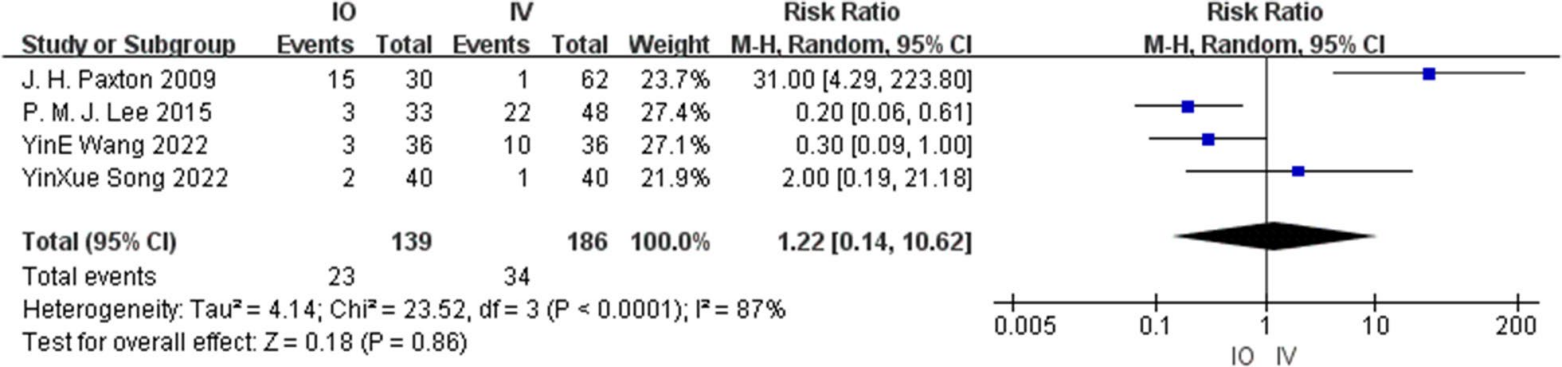
Complications

Six studies [13, 14, 17–20] reported complications, involving 314 patients in the IO group and 375 patients in the IV group. The complications reported included bone injury, soft tissue necrosis, extravasation, osteomyelitis, and osteofascial compartment syndrome. The most common complication was extravasation. Two studies [13, 17] reported that no complications occurred. Heterogeneity (*I*^2^ = 87%) was observed among the studies, and a random-effects model was used. Cumulative analysis showed no difference between the IO and IV group (RR = 1.22, 95% CI [0.14, 10.62], *P* = 0.86). Figure 8 shows a forest plot of the complications.

### Publication bias

**Fig. 9:**
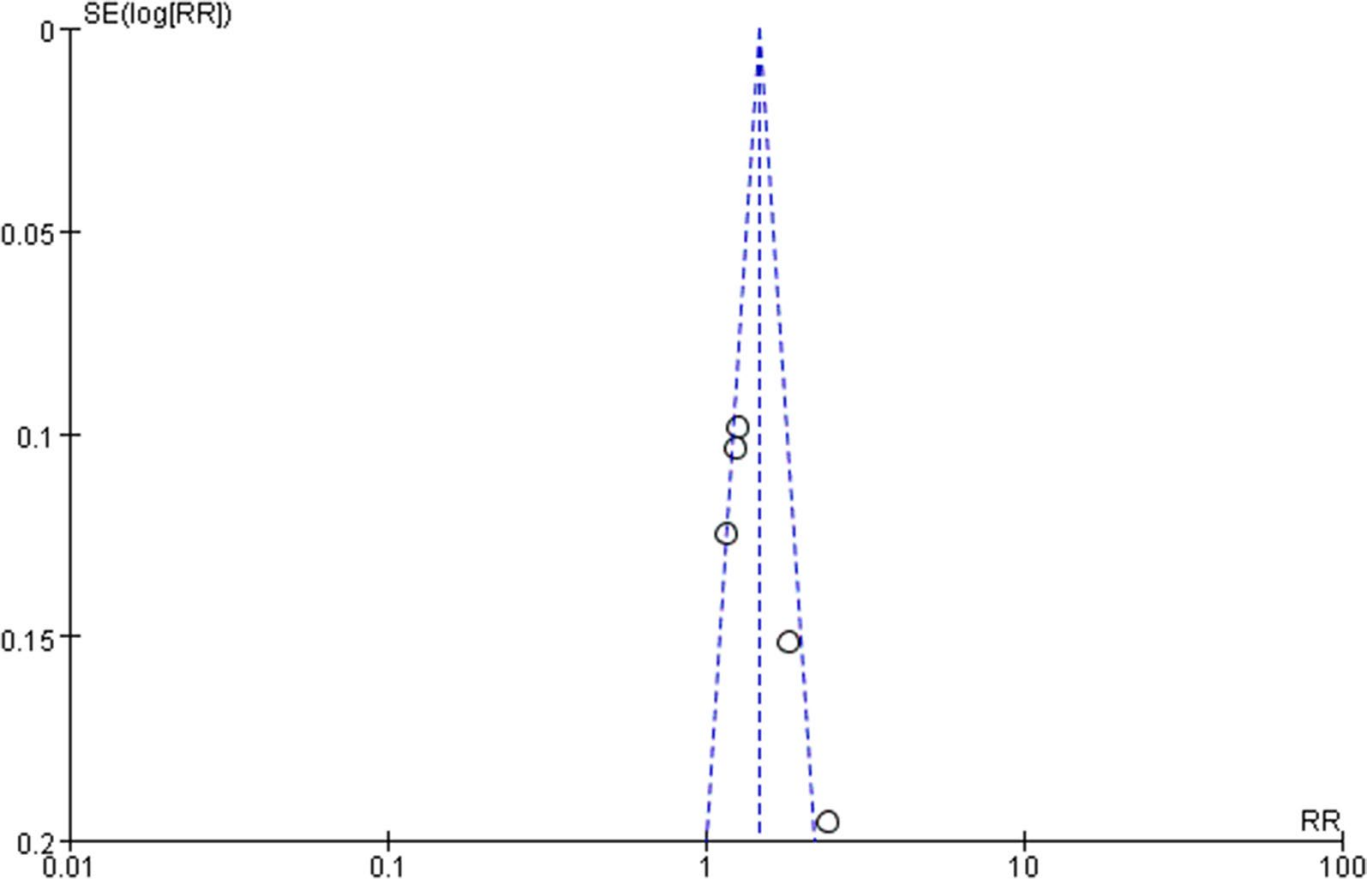
Funnel plot of success rate on first attempt

**Fig. 10:**
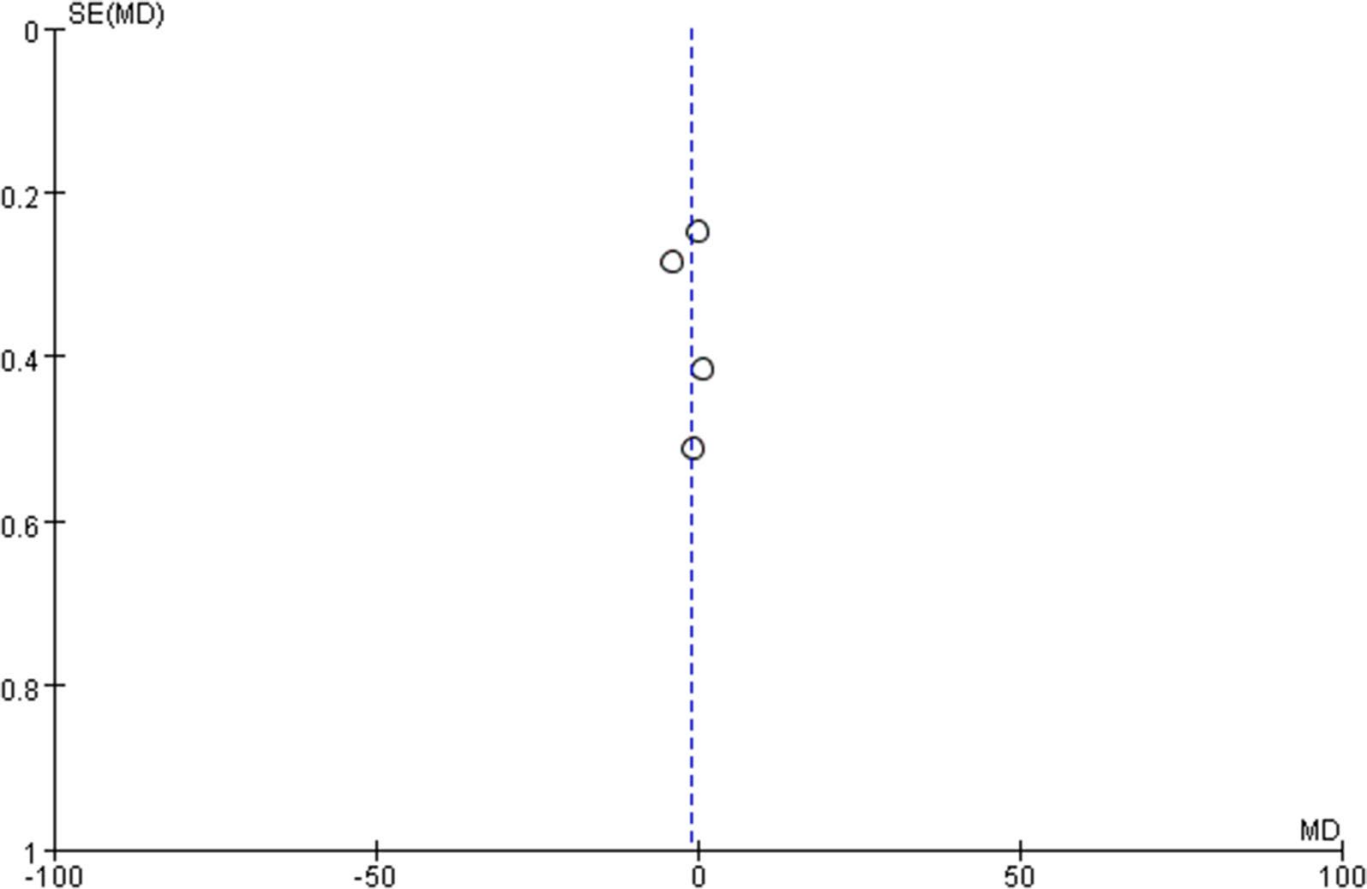
Funnel plot of mean time to resuscitation

**Fig. 11:**
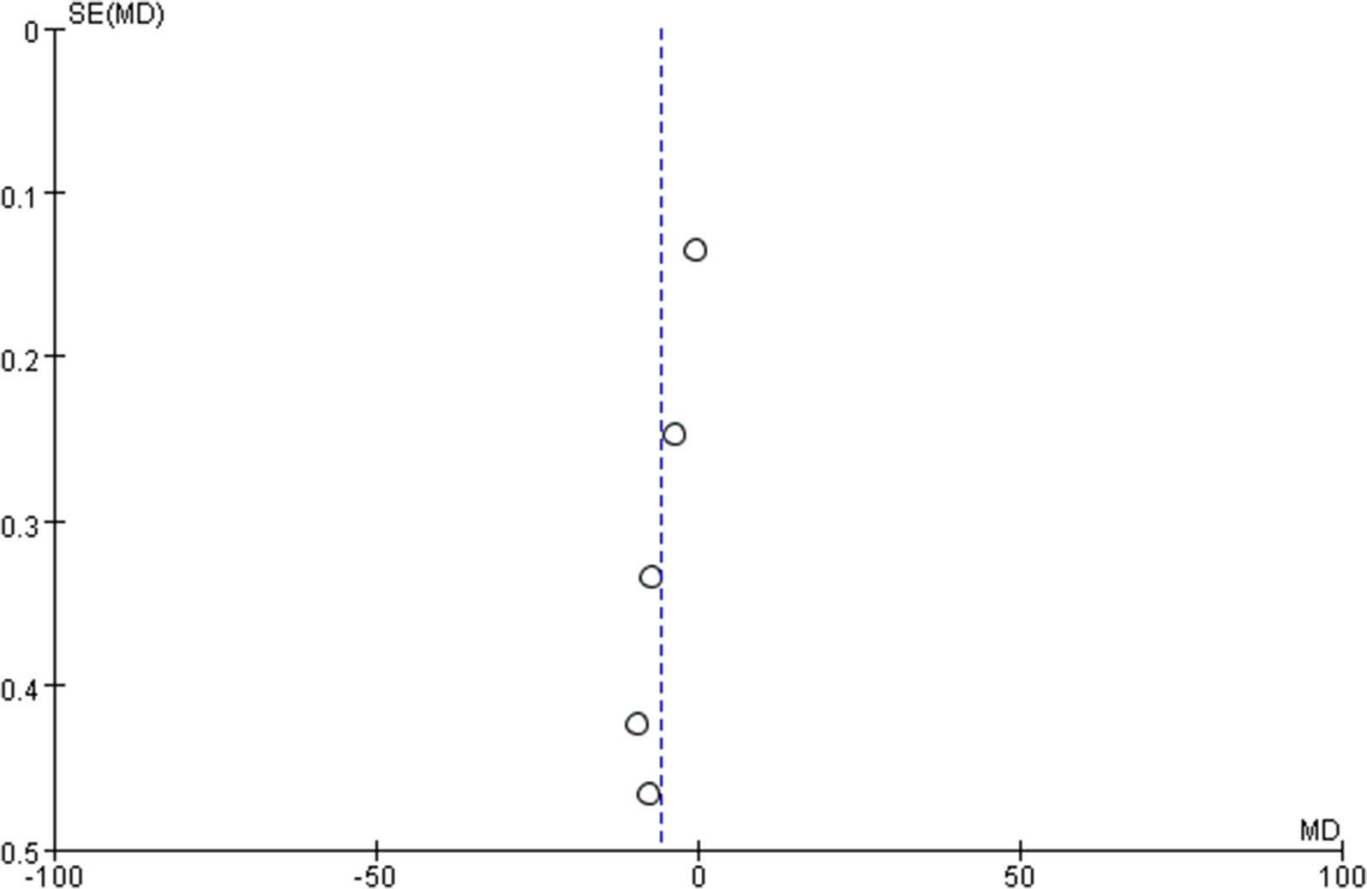
Funnel plot of mean procedure time

**Fig. 12:**
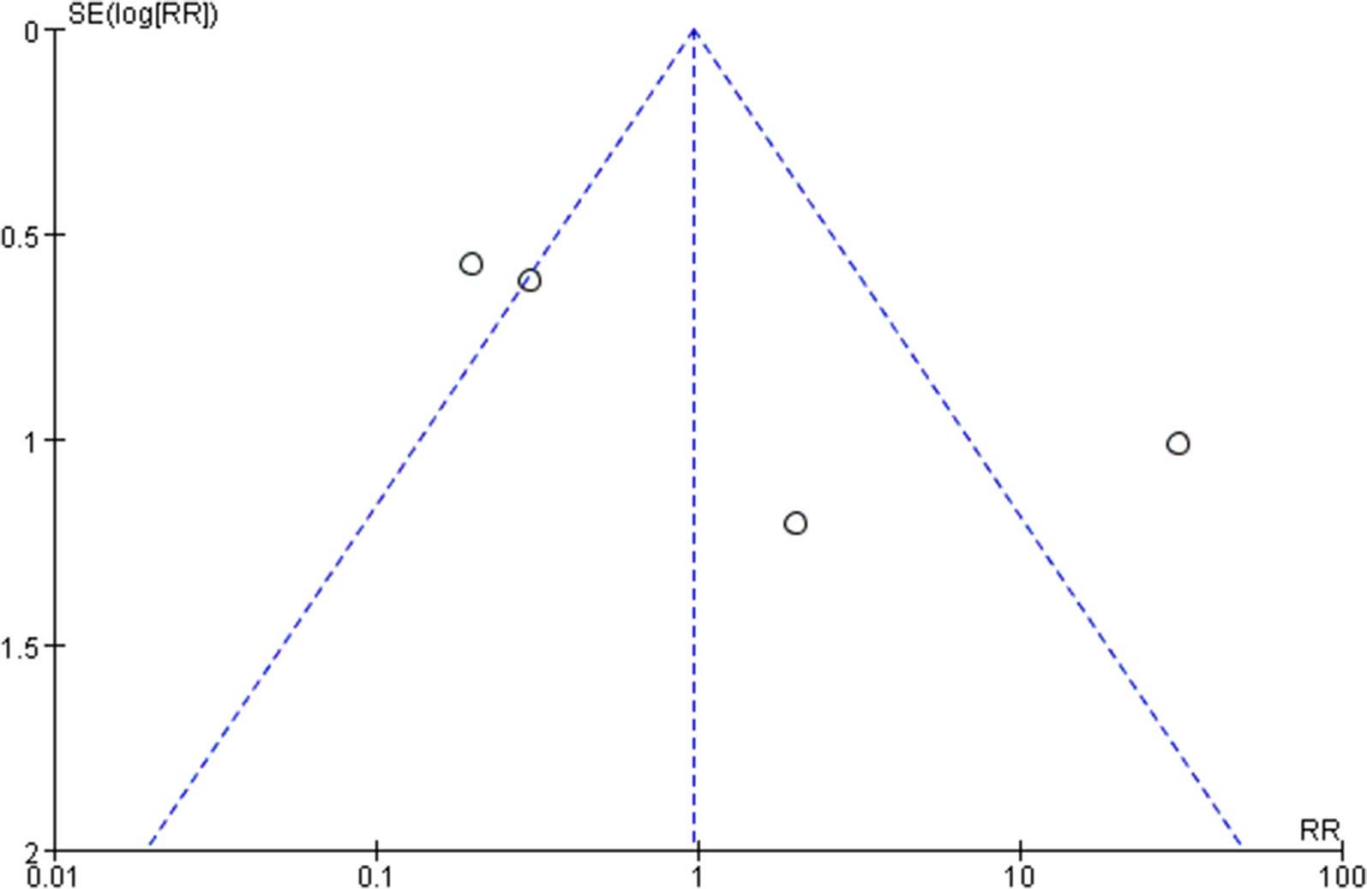
Funnel plot of complications

The funnel plots used to evaluate publication bias were symmetrical, indicating that no publication bias was discovered. Funnel plots of all outcomes are shown in Figs. 9, 10, 11 and 12.

### Sensitivity analyses

To assess the stability of the results, a sensitivity analysis was conducted. Regarding the mean time to resuscitation and mean procedure time, excluding individual studies did not have an impact on the results when compared to the pooled results. Regarding complications, the exclusion of Paxton et al. [14] study changed the results when compared to the main results.

### GRADE summary of evidence table for key outcomes

The GRADE tool was used to evaluate the certainty of evidence. Considering that the number of included studies was limited, that the clinical characteristics of the population in each study were different, there existing the potential for heterogeneity, and that blinding of the surgical intervention was difficult, the results should be interpreted with caution. Given the first-pass success rate and all three secondary outcomes, the quality of evidence was regarded as moderate.

## Discussion

In this systematic review and meta-analysis, we found that the success rate on first attempt of IO access in trauma patients was significantly higher than that of IV access. In addition, the mean procedure time was significantly reduced. Therefore, IO access can help physicians gain efficient vascular access and inject a sufficient amount of liquid into blood vessels in a brief time. During emergency and pre-hospital emergency treatments, most severe trauma patients are in a state of shock, causing vasoconstriction and closed micro-circulation, which makes it difficult for physicians to obtain vascular access and deliver life-saving medications and fluids through peripheral veins. The bone marrow cavity of patients in the shock stage does not collapse [21] and infused fluid can enter the capillaries through the blood sinuses in the bone marrow cavity. Furthermore, it can withstand high pressure and maintain a good perfusion state. Therefore, even if capillaries are frequently closed to the passage of blood, they do not affect the efficiency of IO access. Some studies have shown that the earlier the administration, the better the resuscitation effect. With this consideration in mind, the role of IO vascular access in pre-hospital and emergency environments should be emphasized.

Many factors affect the success of IO puncture, such as puncture sites, IO device, and proficiency level of the operator. The common puncture sites of the IO are the sternum, proximal humerus, and proximal tibia [22]. It has been found that sternal puncture site is easily accessible and is in close proximity to the central venous circulation. Therefore, sternal infusions reach the central circulation faster than infusions in other insertion sites [23]. The humeral site can reach a speed equivalent to that of the sternal puncture site. At the same time, less pain was observed in some studies [24] compared to other sites. However, it may lead to dislodgement and difficulty in identifying anatomical landmarks [25, 26]. In addition, the most popular site for IO access remains the proximal tibia since its first description in 1940 [27]. Improper puncture operation and site may lead to resuscitation failure and even corresponding complications. Many studies have focused on randomized simulation studies of physicians [28]. It was concluded that the success rate may differ considerably between practitioners [29, 30].

The higher success rate on first attempt of IO access and the shorter procedure time can result in faster vascular access and shorten the time of first aid. Maintaining a high flow rate can ensure the effect of resuscitation and administration of medications or fluids. Righi et al. [31] reviewed the flow rate of IO access and revealed that the flow rate through an intraosseous catheter varies widely, depending upon the device used, anatomic insertion site selected, type of medication or fluid being infused, and other features of the infusion kit. Some studies have shown that flow rates with direct venous catheterization are generally higher than that achievable via the IO route, which may be one reason for the preferential use of direct venous access in resuscitative situations [32, 33]. But we also found that the maximum infusion speed of IO is similar to that of IV [22]. Consequently, we hold that the effect of IO is equivalent to that of IV.

Safety is always an important aspect. In this systematic review and meta-analysis, we found that there was no statistical difference in complications between the IO and IV group. The most common types of complications from IO include bone injury, extravasation, osteomyelitis, and compartment syndrome. Besides the inability to insert the needle or a subsequent displacement, the complication rate of IO access has been reported to be lower than 1% [34, 35], which is much lower than that of IV access in adults. With updated equipment, the IO needle has developed into a mechanical device with a higher success rate of puncture, stronger support, and no longer fall off easily, increasing its reliability.

Mechanical IO equipment did not appear until the end of the twentieth century. With the development of technology, newer equipment has been developed or modified from IO needle into various manual devices. There are currently three mechanical IO devices approved by the United States Food and Drug Administration (FDA): the FAST-1 (Pyng Medical Corporation, Vancouver, British Columbia, Canada), the BIG (WaisMed, Yokneam, Israel), and the EZ-IO (Vidacare Corporation Shavano Park, Texas, USA). The BIG and FAST-1 are both spring-loaded, disposable, single-use devices, while the EZ-IO includes a reusable power driver with single-use needle sets. A short cut review carried out in 2011 suggested that traditional manual intraosseous infusion devices have better success rates and faster insertion times compared with semi-automatic intraosseous infusion devices in the prehospital setting [36]. Some studies showed that the EZ-IO demonstrated higher success rates than the BIG, and the BIG could be placed significantly faster than the FAST1 [37, 38]; but others conducted that the differences between both IO devices were not statistically significant [39]. By all accounts these equipment can be placed fast with a high first-attempt success rate [22].

In addition to the convenience and practicality of application, IO access incurs lower financial costs. A study from six centers in U.S. revealed more successful IO catheter placement and few complications. It was estimated that placing IO catheters instead of CVCs in 20% of those cases could represent a savings of $512 million in U.S. related to the cost of treating complications [40].

None of the articles we included provided survival data. Many factors can affect the survival outcomes of patients. Yu-Lin et al. [41] revealed that time to intervention was identified to be an important outcome moderator. To compare the effects of IO and IV access alone, we must exclude other confounding factors. Two previous meta-analyses [41, 42] indicated that IO access was associated with worse survival outcomes for OHCA patients compared with IV access. A likely reason may be that patients who received IO access were more seriously ill. These studies [43–47] of OHCA patients showed that there are significant differences in pre-access characteristics between the IO and IV groups, such as age, sex, witnessed status, and initial shockable rhythm. Some differences did not diminish even after propensity score matching (PSM) [45]. In case of trauma, Smith et al. reported that patients in the IO group were more severely injured with worse outcomes [48]. Another study by Helm et al. reported similar patient characteristics and results [49]. In most cases, IO access is an alternative when an attempt at IV access fails. In addition, the maximum speed of IO access is designed to be much higher than that of IV access; however, some retrospective studies reported a lower speed of infusion via the IO route. This is also a reason for worse outcomes. Most drugs given by IO have been shown to have equal availability and physiologic effect as the same dose given through peripheral IV [22]. By contrast, the pharmacokinetic profile of some drugs can change when administered by IO access or at low doses [50–53]. Considering the differences between the pharmacokinetic parameters of these medications, there is much room for further studies.

Although IO technology has become increasingly popular and medical staff have more knowledge of IO, some studies have shown that the current use of IO is not optimistic. Some questionnaires have indicated that IO was primarily used in emergency departments, but the application frequency varied widely. The main reasons for not using IO were lack of equipment and lack of training. There was also no local guidelines on IO infusion [7–9, 54]. A web-based survey conducted throughout China has demonstrated that 57.4% respondents have heard of intraosseous access, and the most common way to learn about it was from academic conferences. While 10.3% respondents had access to an intraosseous device in their departments, only 6.9% had ever performed intraosseous procedures [55]. Therefore, the more widespread teaching of this technique for emergency use is recommended.

We applied the GRADE tool to evaluate all outcomes. Considering the first-pass success rate and all three secondary outcomes, the results indicated that the strength of the evidence was moderate. Accordingly, although current evidence supports the notion that IO access may benefit trauma patients in pre-hospital care, more rigorous, well-designed studies are still needed to verify the efficacy in future.

### Limitations

The lack of high-quality, large-scale RCTs and the heterogeneity of patients in these retrospective studies are major limitation of our systematic review. The strength of the pooled forces on all four outcomes was moderate. And the survival outcome is not reported by any current study. In addition, a high degree of heterogeneity was observed in meta-analyses of retrospective experiments and in RCTs, which is related to methodological and patient selection heterogeneity in studies. In included articles, the definition of time to resuscitation is not all the same. The study by Paxton et al. reported that measurement of the time to resuscitation began when the skin was sterilized before catheter insertion and ended when the flow of intravenous fluids was subjectively deemed to be adequate for resuscitative purposes, while the other two studies regarded the moment when the blood pressure starts to rise as the end time. This kind of variety of definitions make up one of the major heterogeneities. Considering the low strength of evidence body and high heterogeneity, the results should be explained cautiously, and further large scale, long-term, high-quality RCTs or prospective cohort studies are needed to explore the effectiveness of IO treatment.

## Conclusions

Our systematic review and meta-analysis indicate that the success rate on first attempt of IO access was much higher than that of IV access for trauma patients, and the mean procedure time was significantly reduced. Therefore, IO access should be suggested as an urgent vascular access for hypotensive trauma patients, especially those who are under severely shock. However, the evidence is not strong enough. The evaluation of the strength of the evidence level of major outcomes using the GRADE tool indicated that the level of evidence of these outcomes was moderate. Thus, more rigorously designed large-scale clinical trials are urgently needed to evaluate the best scenario for IO access in emergency department and prehospital care.

## Supplementary Information

**Additional file 1.** The search strategy performed in the databases.

## Data Availability

All data generated or analyzed during this study are included in this published article (shown in references 13–20).
