# Supplementary material for: Efficacy of intraosseous access for trauma resuscitation: a systematic review and meta-analysis
Source: World J Emerg Surg. 2023 Mar 14;18:17. doi: 10.1186/s13017-023-00487-7 (PMC10012735; doi:10.1186/s13017-023-00487-7)
Supplement: Supplementary file 1 — Additional file 1. The search strategy performed in the databases. [file 13017_2023_487_MOESM1_ESM.docx]

**eTable**
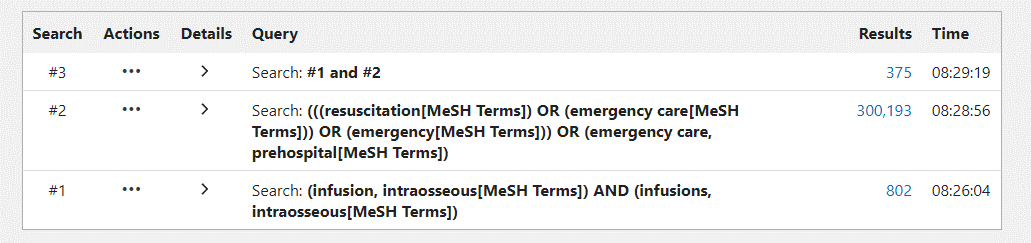
 **1.** **Search strategy performed in the PubMed database**


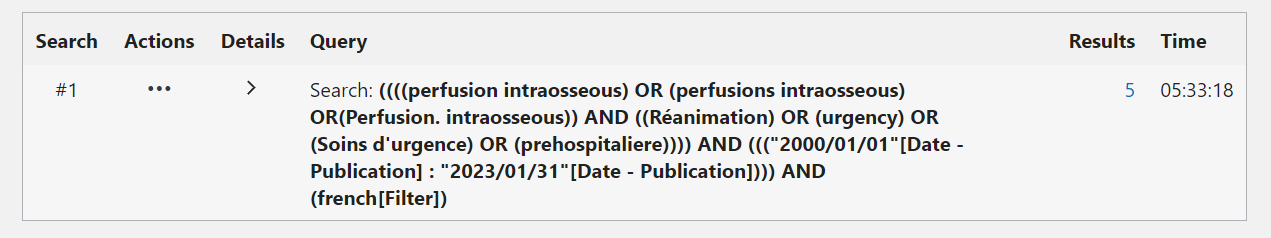


**eTable 2.** **Search strategy performed in the banque de données en santé publique (BDSP) database**

**
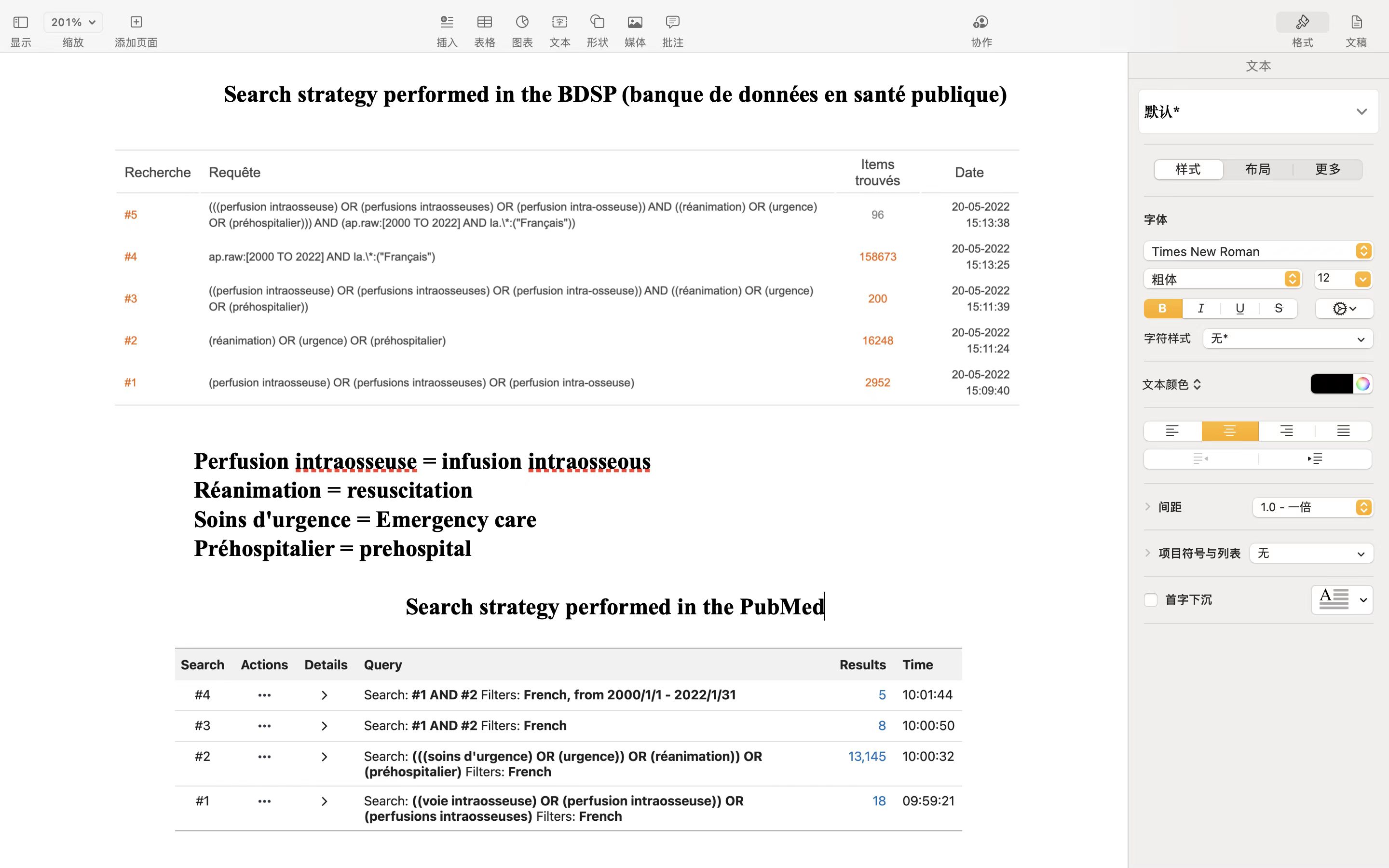
**

**eTable 3.** **Search strategy performed in the ScienceDirect database**

**
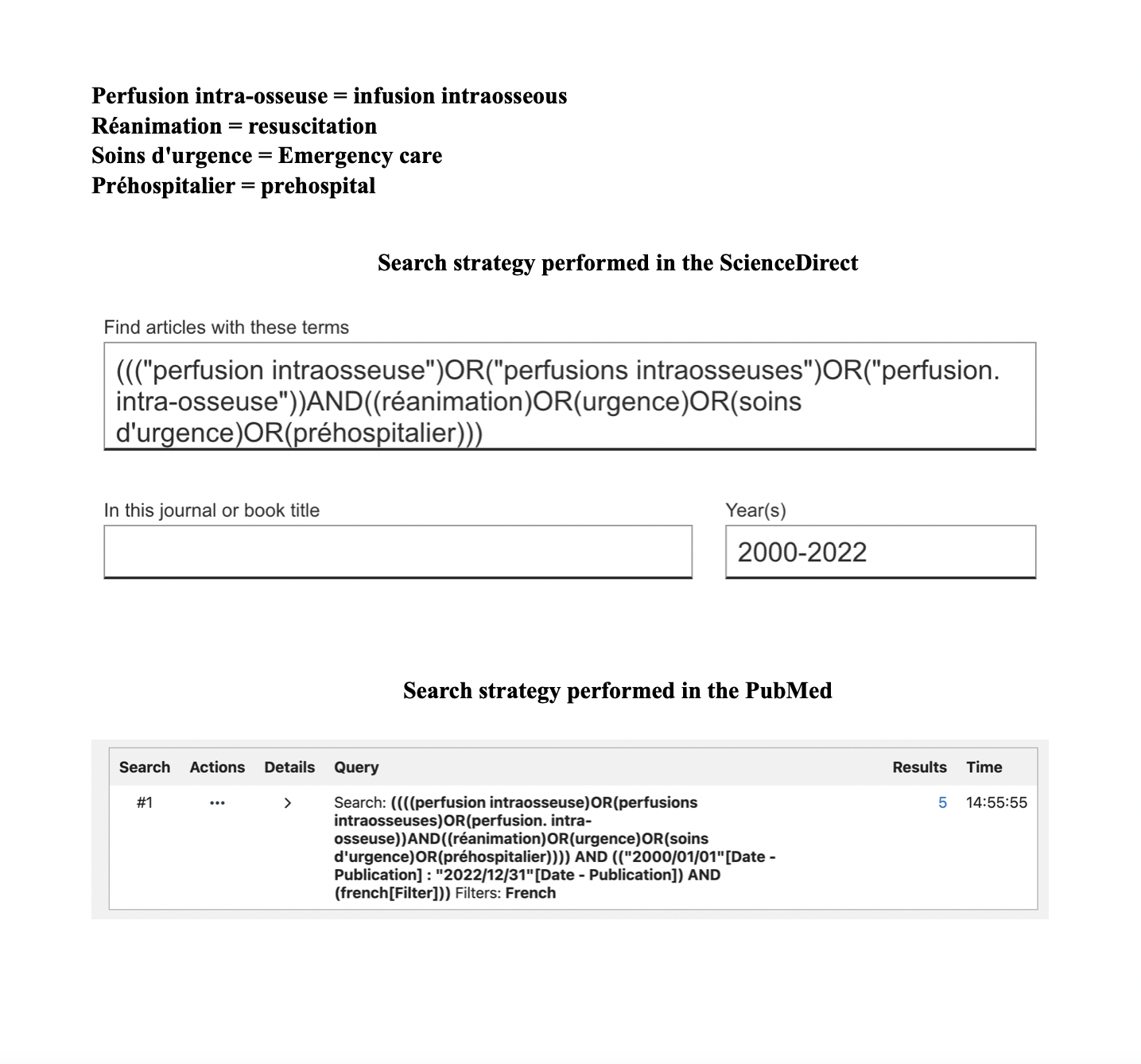
**

Perfusion intraosseuse = infusion intraosseous; Réanimation = resuscitation; Soins d'urgence = Emergency care; Préhospitalier = prehospital
